# Variations in oral microbiota associated with oral cancer

**DOI:** 10.1038/s41598-017-11779-9

**Published:** 2017-09-18

**Authors:** Hongsen Zhao, Min Chu, Zhengwei Huang, Xi Yang, Shujun Ran, Bin Hu, Chenping Zhang, Jingping Liang

**Affiliations:** 1 Ninth People’s Hospital, School of Medicine, Shanghai Jiao Tong University, Department of Endodontics and Operative Dentistry, Shanghai Key Laboratory of Stomatology, Shanghai, 200011 China; 2 Ninth People’s Hospital, School of Medicine, Shanghai Jiao Tong University, Department of Prosthodontics, Shanghai, 200011 China; 3 Ninth People’s Hospital, School of Medicine, Shanghai Jiao Tong University, Department of Oral Maxillofacial-Head and Neck Oncology, Shanghai, 200011 China

## Abstract

Individual bacteria and shifts in microbiome composition are associated with human disease, including cancer. To unravel the connections underlying oral bacterial dysbiosis and oral squamous cell carcinoma (OSCC), cancer lesion samples and anatomically matched normal samples were obtained from the same patients. We then profiled the bacteria within OSCC lesion surface samples at the species level using next-generation sequencing to comprehensively investigate bacterial community composition and functional genes in these samples. Significantly greater bacterial diversity was observed in the cancer samples than in the normal samples. Compared with previous studies, we identified many more taxa demonstrating remarkably different distributions between the groups. In particular, a group of periodontitis-correlated taxa, including *Fusobacterium, Dialister, Peptostreptococcus, Filifactor, Peptococcus, Catonella* and *Parvimonas*, was significantly enriched in OSCC samples. Additionally, several operational taxonomic units (OTUs) associated with *Fusobacterium* were highly involved in OSCC and demonstrated good diagnostic power. Our study revealed drastic changes in surface bacterial communities of OSCC. The findings enrich knowledge of the association between oral bacterial communities and oral cancer.

## Introduction

The human body is inhabited by over 100 trillion microbial cells living in symbiosis with their host^1^. Bacteria at certain body sites have long been believed to be involved in immune modulation, disease development, and health maintenance. The term microbiome was coined to describe “the collective genomes and gene products of all microbes residing within an organism”^2^. With the advent of high-throughput, next-generation sequencing (NGS), there has been a surge of interest in studying the human microbiome in the context of disease. Recent studies have demonstrated the importance of the gut microbiota in digestion, fat storage, angiogenesis, immune system development and responses, resistance to colonization, epithelial architecture^3,4^, and dysbiosis, which is believed to contribute to the pathogenesis of local and systemic diseases, including inflammatory bowel disease, diabetes, and colorectal cancer^5^. Located at the beginning of the aerodigestive tract, approximately 700 prokaryote species have been detected in the human oral cavity. These species belong to 185 genera and 12 phyla, of which approximately 54% are officially named, 14% unnamed (but cultivated) and 32% known only as uncultivated phylotypes^6^. This oral bacterial flora plays an essential role in maintaining a normal oral physiological environment and is associated with host health^7^. In contrast to traditional views, recent analyses suggest the involvement of a consortium of microbes, rather than a single species, as causing disease^8^, a phenomenon that has been well characterized for periodontal diseases^9^.

Oral cancer, primarily oral squamous cell carcinoma (OSCC) deriving from the oral mucosa, is a disease that arises from both host genetics and environmental factors; tobacco and alcohol consumption, betel quid chewing, and human papillomavirus infection are well-known risk factors^10^. The incidence of oral cancer is increasing, and this disease continues to be a major global health problem. Furthermore, approximately 15% of oral cancer cases cannot be attributed to the aforementioned major risk factors, resulting in the need to explore other potential risk factors^11^. A plethora of bacteria, the proverbial bacterial biofilm, coat each surface of the oral cavity^12^, and groups inhabiting the mucosal surface might constitute the bulk of the tumor microenvironment. To date, various microbes and changes in different bacteria have been associated with several types of cancer^13^. Cancer-associated changes in the oral microbiome have been assessed in several early studies employing culture-based or molecular techniques^14–19^, but a consensus has not been reached due to the limited number of strains/clones that it is feasible to test. However, the emergence of NGS allows microbial communities to be profiled at an unprecedented depth and coverage.

To date, several studies have employed NGS to assess bacterial profiles associated with OSCC. Pushalkar *et al*. evaluated the diversity and relative abundance of bacteria in the saliva of subjects with OSCC; however, only three OSCC cases and two healthy controls were included^20^. Later, a larger-scale study analyzed swabs of lesion surfaces and contra-lateral normal mucosae from 18 OSCC patients, and significant decreases in the abundances of the genera *Firmicutes* and *Actinobacteria* were observed in cancer samples^21^. In another report, saliva bacterial communities in six OSCC patients were elucidated by performing pyrosequencing, and paired taxa within the family *Enterobacteriaceae* together with the genus *Oribacterium* were suggested to distinguish OSCC samples from oropharyngeal squamous cell carcinoma (OPSCC) and normal samples^22^. Regardless, the studies above failed to provide bacterial composition at the species level, even though specific species or even strains are usually involved in disease. Al-hebshi successively profiled bacterial communities within 23 OSCC tissue samples from Yemeni patients at the species level, providing the first epidemiological evidence for associations of *Fusobacterium nucleatum* and *Pseudomonas aeruginosa* with OSCC^23,24^. Given the limited number of OSCC samples included, the significance of the findings from these studies is unclear. Accordingly, more studies are warranted to validate these results.

In the current study, cancer lesion samples and matched controls were procured from 40 Chinese subjects with OSCC. Bacterial profiles within the samples were characterized at the species level. Shifts in bacterial composition and gene functions associated with OSCC were described and analyzed. In particular, we detected a group of periodontitis-related taxa that was significantly enriched in OSCC samples. Our findings may contribute to further clarification of the connection between OSCC and oral bacteria.

## Results

### Overall structure of bacterial communities across samples

**Figure 1:**
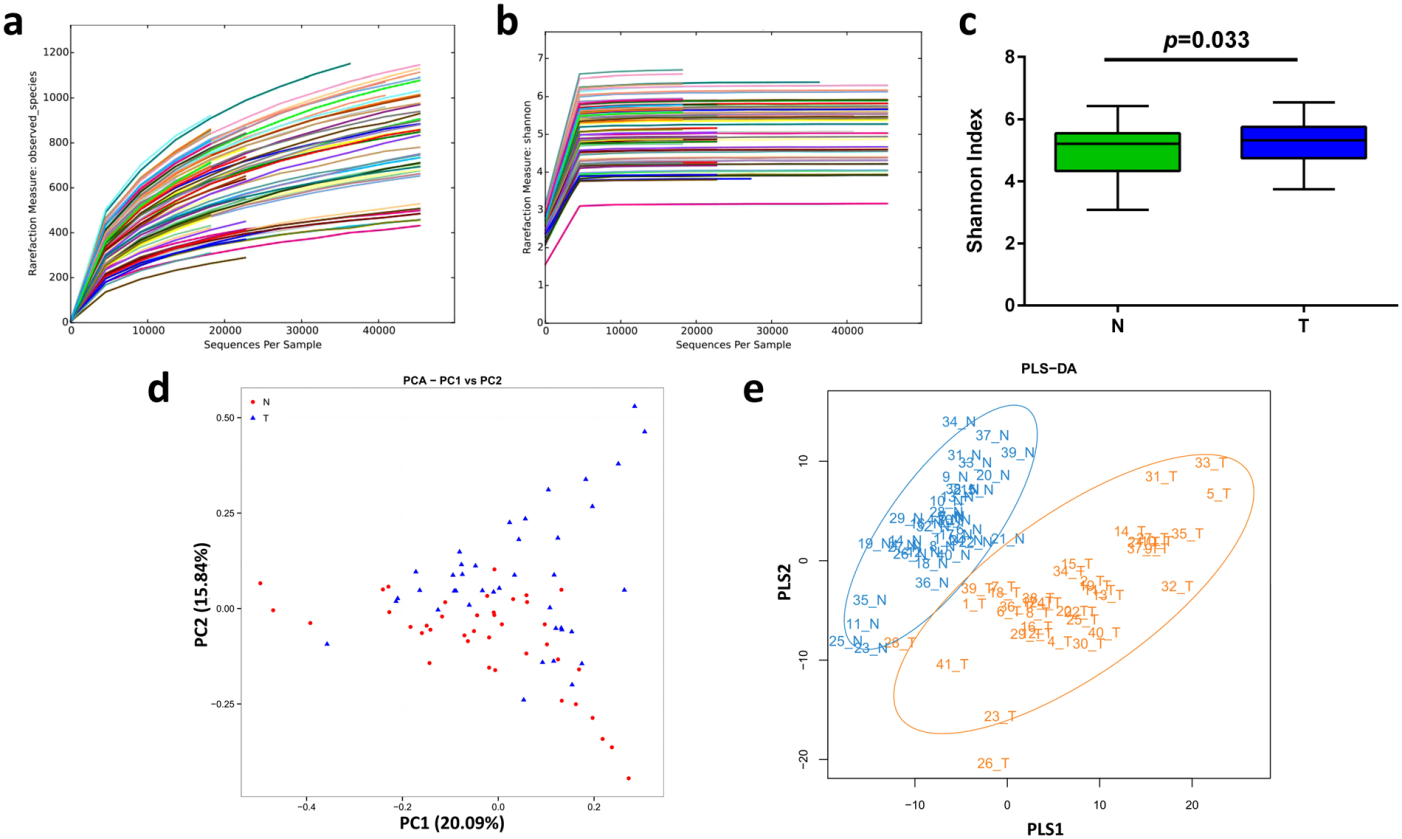
Comparison of oral microbiota structures in the N and T groups. (**a**) Rarefaction analysis of bacterial 16 S rRNA gene sequences was performed to evaluate whether further sequencing would likely detect additional taxa, indicated by a plateau. Different colors represent different samples. (**b**) Shannon index curves were constructed to evaluate the numbers of samples likely required to identify additional taxa, indicated by a plateau. Different colors represent different samples. (**c**) Box plots depict differences in bacterial diversity between the N and T groups according to the Shannon index. (**d**) PCA at the OTU level. (**e**) Partial least square discriminant score plot of oral microbiota between the N and T groups. N, clinical normal samples; T, oral cancer samples.

In the current study, 80 samples were sequenced using an Illumina MiSeq system, and a total of 5,075,391 raw sequences were generated. After quality trimming and chimera checking, 4,075,169 high-quality sequences with an average length of 391 bp were recovered for downstream analysis, with an average of 50,940 reads (ranging from 19,353 to 117,244 reads) per sample. After alignment in the HOMD, unique representative sequences were classified into 2,334 operational taxonomic units (OTUs) at a 97% similarity level, from which 11 phyla, 130 genera and 389 species were detected. Good’s estimator of coverage was 99.26%, indicating the 16 S rRNA sequences identified in this study likely represent the majority of bacterial sequences present in the samples. Different indexes (Shannon, Simpson, Abundance-based Coverage Estimator (ACE), and Chao 1) were employed to estimate the α-diversity of the bacterial community. Although rarefaction curves of numbers of observed OTUs per sample suggested new phylotypes would be expected with additional sequencing (Fig. 1a), the rarefaction curves for the Shannon diversity index for each sample reached plateaus, indicating that the majority of the diversity was already procured (Fig. 1b). As revealed by the Shannon diversity index (Fig. 1c), the diversity of the bacterial community in the cancer samples was significantly increased compared with that of the corresponding clinically normal control samples. A similar trend was observed when employing other diversity indexes (Simpson, ACE, Chao 1), although without statistical significance (Supplementary Fig. S1).

To evaluate the extent of the similarity of the bacterial communities, unweighted UniFrac Principal Component Analysis (PCA) at the OTU level was employed and indicated no obvious separation between groups (Fig. 1d). Then, Partial Least Squares Discriminant Analysis (PLS-DA), a supervised analysis suitable for high-dimensional data, was performed (Fig. 1e). The bacterial communities in the cancer samples and the matched controls clustered separately, suggesting the overall structures of the bacterial communities in the groups were significantly different. Spots representing the cancer samples presented more dispersed distribution patterns than those of the controls, aligning with the increased level of bacterial diversity found in the cancer samples. A nonparametric multivariate analysis of variance (Adonis) and an analysis of similarities (ANOSIM) based on UniFrac distances were performed, and the calculated P values (P = 0.002 for Adonis, P = 0.001 for ANOSIM) further demonstrated the remarkable differences between the bacterial communities in the groups.

### Common and distinct bacterial taxa in the analyzed groups

**Figure 2:**
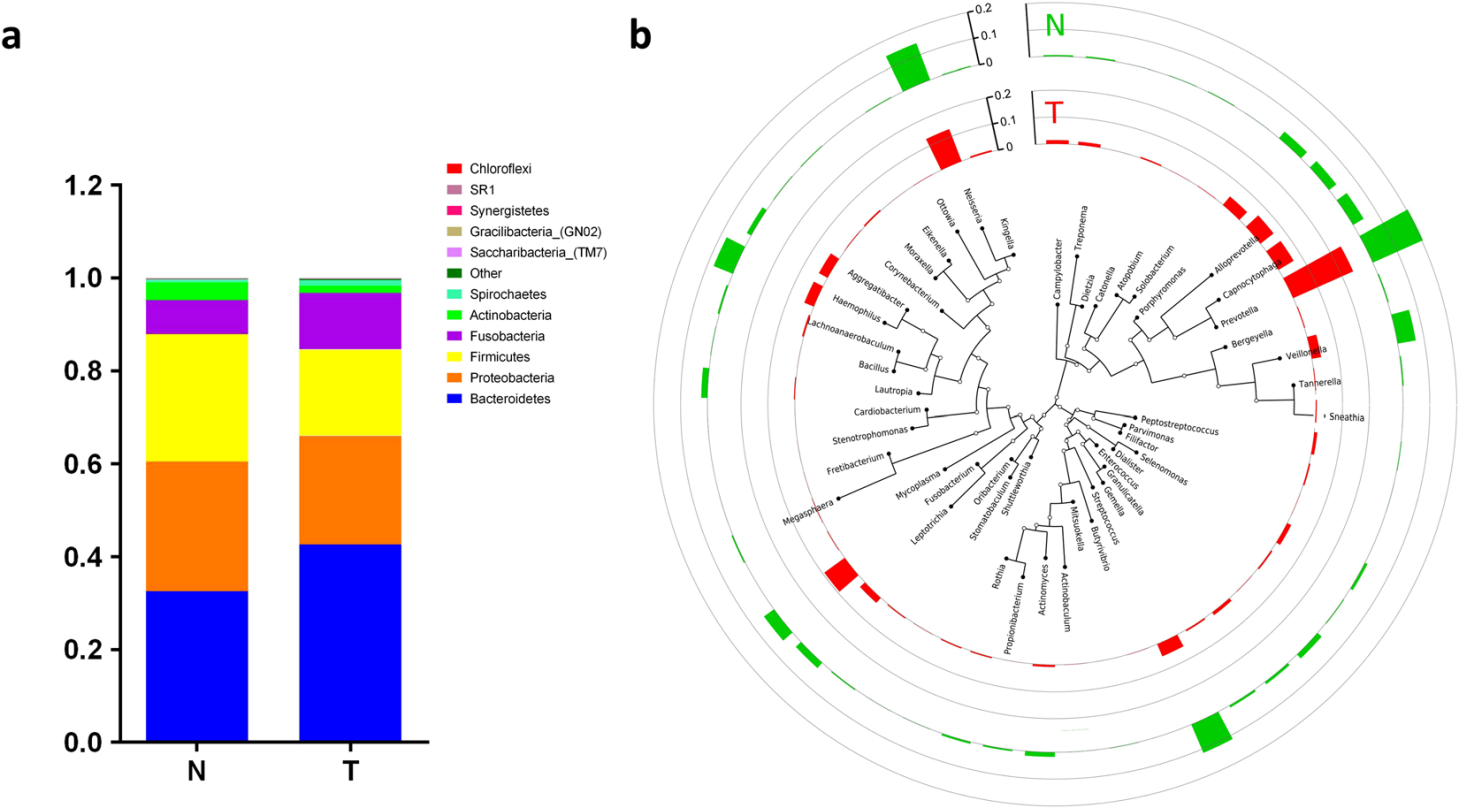
Composition of bacterial communities across samples at the phylum and genus levels. (**a**) Relative abundance of bacterial phyla among the N and T groups. (**b**) Classification tree of the 50 most abundant genera across all samples. The outer cycle colored bars represent the relative abundances of taxa in each group. N, clinical normal samples; T, oral cancer samples.

The bacterial communities in the cancer lesions and the controls were analyzed at different taxonomic levels (Fig. 2 and Supplementary Fig. S2). *Bacteroidetes, Proteobacteria, Firmicutes, Fusobacteria, Actinobacteria*, the top five most abundant phyla, together comprised 98.62% of all sequences (Fig. 2a). *Bacteroidetes* was the most abundant phylum, accounting for 37.6% of sequences. In contrast, the abundances of the other detected phyla, including *Synergistetes*, *SR1*, and *Chloroflexi*, were less than 0.1%. At the genus level, *Prevotella*, *Neisseria, Streptococcus, Fusobacterium*, and *Haemophilus* were the five most abundant genera, comprising 22.46%, 13.67%, 8.17%, 6.95%, and 5.74% of sequences, respectively (Fig. 2b). Of all genera detected, 18 taxa were found in all samples; in addition to the five genera mentioned above, these included *Capnocytophaga, Veillonella, Alloprevotella, Porphyromonas, Leptotrichia, Aggregatibacter, Selenomonas, Campylobacter, Granulicatella, Actinomyces, Gemella, Lachnoanaerobaculum*, and *Bergeyella*. The shared genera collectively represented over 80.0% of all detected sequences. At the species level, an average of approximately 200 species were detected per sample. The relative abundances of eleven species each surpassed 2%; specifically, *Neisseria flavescens, Prevotella melaninogenica, Fusobacterium periodonticum, Streptococcus oralis, Prevotella intermedia, Veillonella atypica, Haemophilus parahaemolyticus, Porphyromonas sp. _oral_taxon_279, Capnocytophaga leadbetteri, Alloprevotella sp. _oral_taxon_473* and *Haemophilus parainfluenzae* together accounted for a total of 53.8% of sequences (Supplementary Fig. S2). *Neisseria flavescens* was the most abundant species in both groups, with a relative abundance of 10.73% in cancer lesions and 12.19% in normal controls. Despite significant inter-individual variation, 14 species were detected across all samples, including *Actinomyces odontolyticus, Prevotella melaninogenica, Prevotella scopos, Capnocytophaga gingivalis, Gemella sanguinis, Granulicatella adiacens, Streptococcus oralis, Streptococcus salivarius, Lachnoanaerobaculum umeaense, Veillonella atypica, Fusobacterium periodonticum, Neisseria elongata, Neisseria flavescens*, and *Haemophilus parainfluenzae*, and constituted the oral mucosal core bacteriome of the OSCC patients.

**Figure 3:**
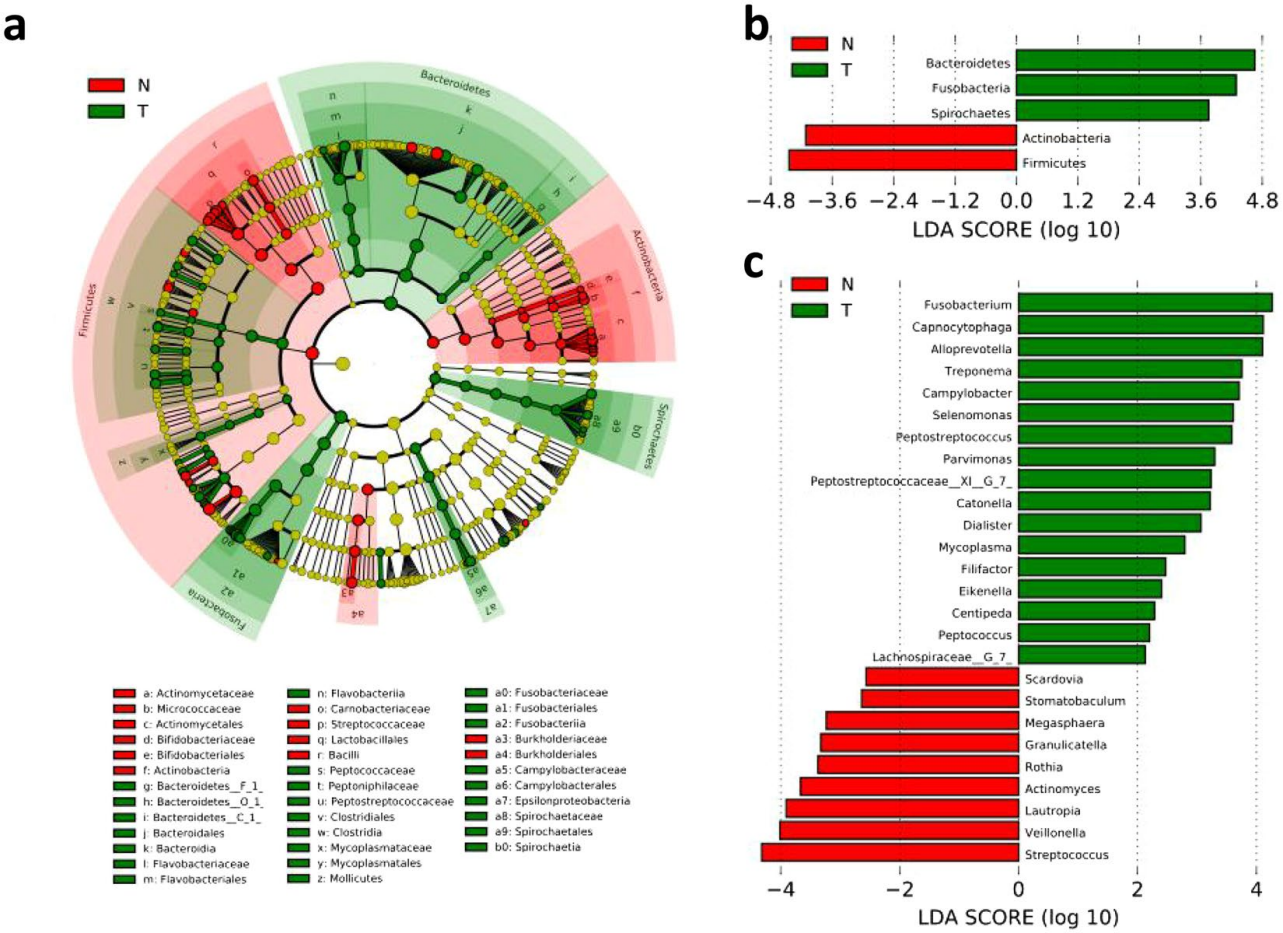
Distinct taxa identified in the N and T groups using LEfSe analysis. (**a**) Cladogram constructed using the LEfSe method to indicate the phylogenetic distribution of bacteria that were remarkably enriched in the N and T groups. (**b**) LDA scores showed significant bacterial differences within groups at the phylum level.(**c**) LDA scores showed significant bacterial differences within groups at the genus level. N, clinical normal samples; T, oral cancer samples.

The bacterial composition of the cancer samples varied from that of the controls. There were 6 phyla and 68 genera with higher abundances in the cancer samples than in the controls. To identify the distinguishing taxa within the groups, the linear discriminant analysis (LDA) effect size (LEfSe) method was implemented (Fig. 3). At the phylum level, *Spirochaetes*, *Fusobacteria*, and *Bacteroidetes* were significantly enriched in diseased samples, while *Firmicutes* and *Actinobacteria* were significantly decreased (Fig. 3b). At the genus level, 17 taxa exhibited significantly higher abundances in the cancer samples than in the controls, including *Mycoplasma, Treponema, Campylobacter, Eikenella, Centipeda, Lachnospiraceae_G_7, Alloprevotella, Fusobacterium, Selenomonas, Dialister, Peptostreptococcus, Filifactor, Peptococcus, Catonella, Parvimonas, Capnocytophaga*, and *Peptostreptococcaceae_XI_G_7*. The taxa *Megasphaera, Stomatobaculum, Granulicatella, Lautropia, Veillonella, Streptococcus, Scardovia, Rothia*, and *Actinomyces* were remarkably prevalent in the controls (Fig. 3c). *Prevotella* and *Neisseria* were the predominant genera in both groups: there was no significant difference in distribution between the two groups (Fig. 2b). At the species level, 39 species were significantly increased, while 28 species were significantly decreased in diseased samples compared to those levels in the controls (Supplementary Fig. S3).

### Co-occurrence network analysis and function predictions

**Figure 4:**
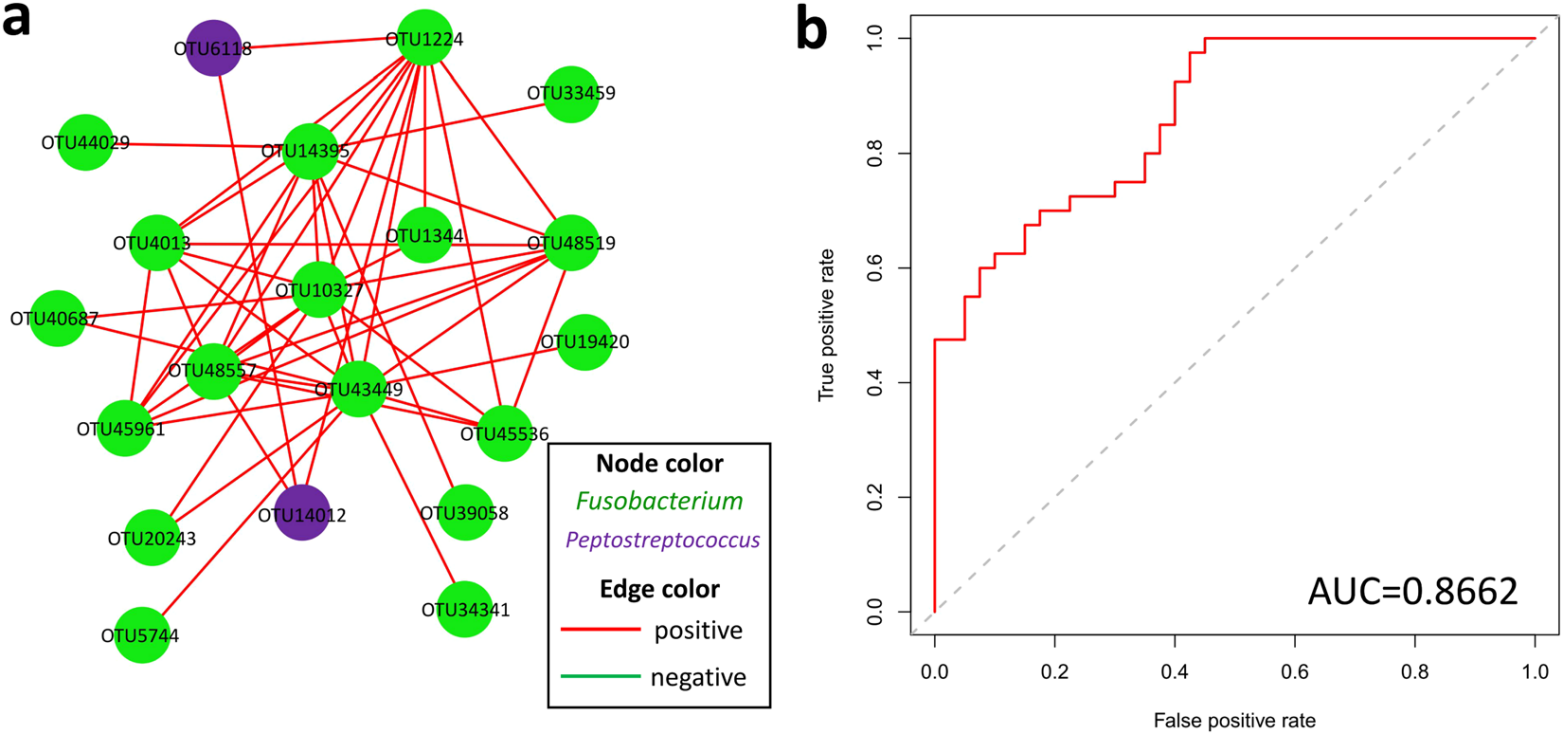
Co-occurrence network of *Fusobacterium* comprising OTUs and the diagnostic power of selected OTUs. (**a**) Each node represents an OTU colored for its genus-level phylotypes, and each edge represents a significant co-occurrence relationship colored according to its association (red: positive, green: negative). (**b**) ROC curves for selected *Fusobacterium* OTUs were constructed to predict diagnostic power.

To predict the ecological relationships across different bacterial communities, spatial Pearson’s correlations between bacterial species were visualized and then analyzed (Supplementary Fig. S4). In the network, pairwise relationships were represented by edges connecting two nodes. There were 1,574 associations among 306 nodes in the controls and 1,794 associations among 366 nodes in the lesions at the OTU level, indicating increased network complexity in the latter group. The genera *Prevotella* and *Neisseria* comprised the two densest clusters in both groups. The hub OTU with the most associations in the clusters, OTU50445 (belonging to *Prevotella melaninogenica*), primarily represented the genus *Prevotella* in both groups, while the hub OTUs in the clusters primarily representing the genus *Neisseria* were OTU52958 in the controls and OTU46085 in the cancer samples, both of which belonged to *Neisseria flavescens*. There was a highly connected bacterial cluster of *Fusobacterium* comprising OTUs (OTU48557, 45961, 43449, 14395, 1224, 10327, and 4013) that were heavily involved in the bacterial ecology structure of the diseased samples compared with the controls, suggesting that *Fusobacterium* may play a critical role in the development of oral cancer (Fig. 4a). The predictive power of the seven described OTUs was further assessed by constructing a receiver operating characteristic (ROC) curve, a function of the true-positive rate (TPR or sensitivity) and false-positive rate (FPR or 1-specificity). The area under the ROC curve (AUC) reached 0.866, indicating good diagnostic performance (Fig. 4b).

**Figure 5:**
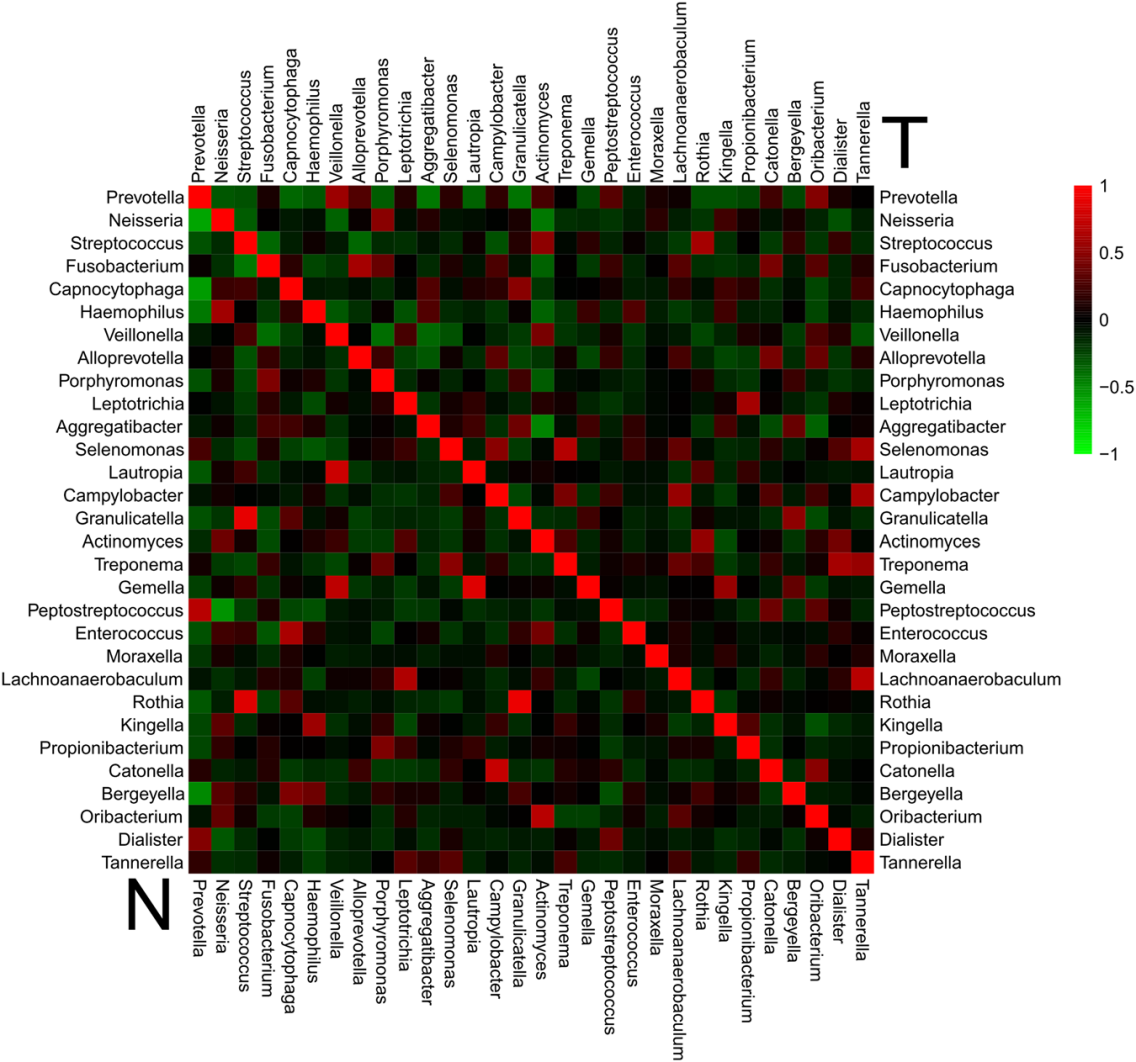
Co-occurrence and co-exclusion analysis of bacterial genera. Pearson correlations among the top 30 most abundant bacterial genera were calculated and analyzed; groups are shown on the left and right. Correlation values ranged from −1.00 (green) to 1.00 (red).

The co-occurrence patterns of the 30 most abundant bacterial genera in each group were investigated in detail (Fig. 5). In matched normal controls, *Tannerella* and *Lachnoanaerobaculum* were the most positively correlated (ρ = 0.681), whereas *Actinomyces* and *Aggregatibacter* were the most negatively correlated (ρ = −0.462). In the cancer samples, the most positively correlated genera were *Rothia* and *Granulicatella* (ρ = 0.903), and *Neisseria* and *Prevotella* were the most negatively correlated (ρ = −0.618). Additionally, opposing co-occurrence patterns at the genus and species level were observed. For example, the genera *Actinomyces* and *Neisseria* correlated positively (ρ = 0.383) in the lesions but were negatively correlated (ρ = −0.429) in normal controls. *Prevotella pallens* and *Prevotella intermedia* demonstrated negative correlations (ρ = −0.222) in lesions but positively correlated in the control group (ρ = 0. 342) (Supplementary Fig. S5).

**Figure 6:**
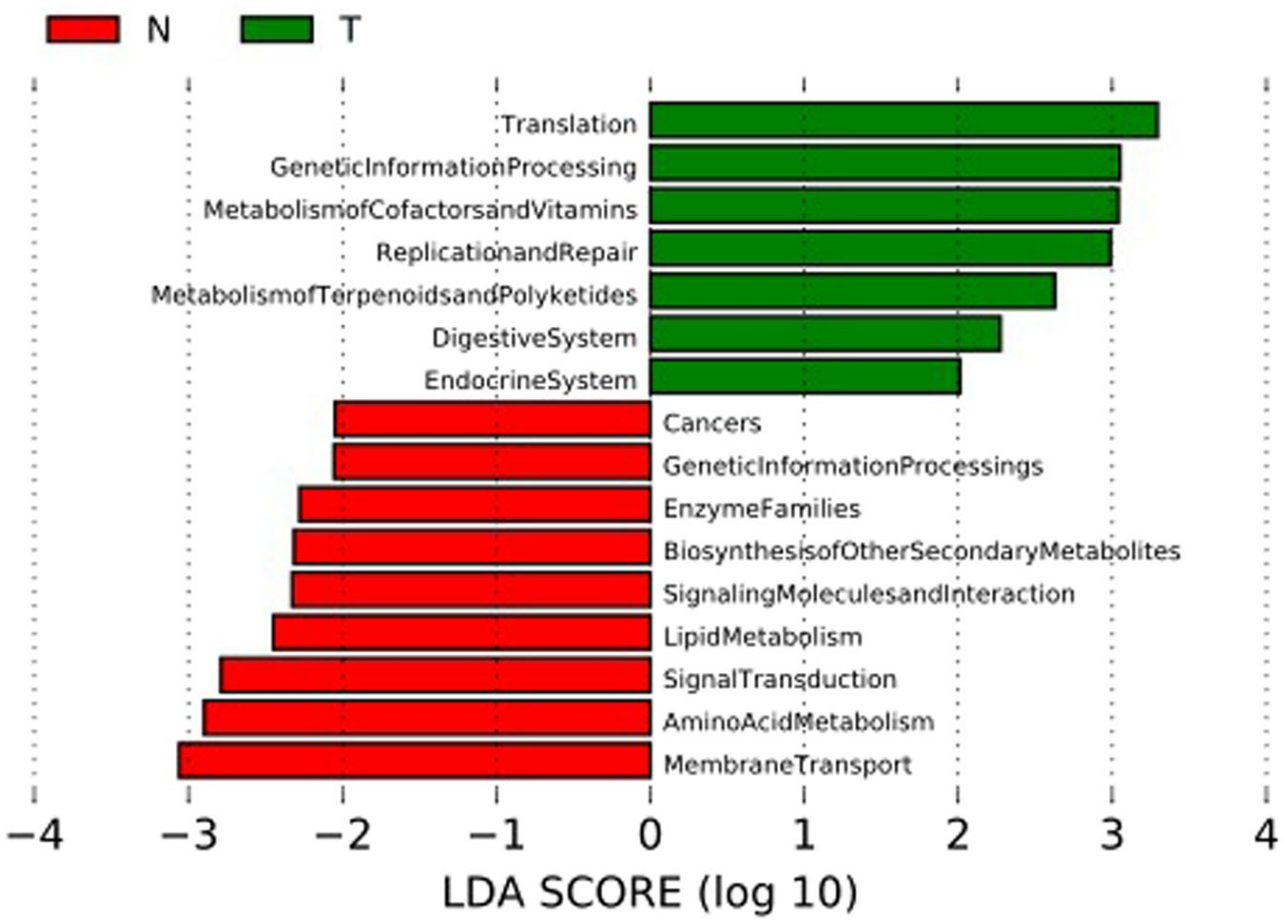
LDA scores predict gene function enriched in different groups using PICRUSt. N, clinical normal samples; T, oral cancer samples.

Finally, the Phylogenetic Investigation of Communities by Reconstruction of Unobserved States (PICRUSt) algorithm was employed to predict bacterial functions in the two groups. The LEfSe outputs showed a series of metabolic pathways presenting significantly different distributions in each group (Fig. 6). Pathways related to Genetic Information Processing were remarkably enriched in cancer lesions.

## Discussion

Interest in a possible relationship between bacteria and different stages of cancer development has been increasing since the classification of *Helicobacter pylori* as a definite carcinogen by the World Health Organization^25^. Although microorganisms have been implicated in 15.4% of human malignancies^26^, there is a paucity of knowledge regarding the role of bacteria in the progression of OSCC. Because bacterial composition at the species level within OSCC samples has rarely been reported, we attempted to add knowledge regarding this aspect.

Compared with the pyrosequencing applied in previous studies^20–23^, Illumina Miseq is a popular established platform of NGS due to its reduced run times and lower cost of reagents, with an overall error rate below 1%, and the 2 × 300 bp read length is flexible for sequencing of small genomes^27,28^. However, the different sequencing regions (V4-V5^20^, V4^21^, V3-V5^22^, V1-V3^23,24^) targeted between studies, along with differences in sample types, selection of control tissues, and number of samples included, may produce inconsistent results. In this study, the V4-V5 region was chosen for sequencing, as it is proposed to be an ideal target for bacteria in 16 S rRNA-based analyses^29^. In the current study, we obtained many more raw sequences and OTUs than those obtained in earlier studies^21,23^, making it possible to comprehensively profile bacterial structure in cancer lesions. Then, we compared the complexity of the bacterial communities present at mucosal sites in both groups, and our results unraveled critical yet pronounced bacterial characteristics associated with disease. Different from previous reports in which tissue biopsies or swabs were analyzed^17,19,21,24^, greater phylogenetic diversity was observed in the OSCC lesion surface in our study, as suggested by rarefaction curves for the Shannon diversity index. With regard to saliva samples, Guerrero-Preston reported a significant loss in richness and diversity of oral bacterial species in oral cancer patients compared to controls^22^, whereas another study revealed much greater diversity of bacterial communities in OSCC samples^30^. Moreover, Adonis and ANOSIM analyses further corroborated the significant differences between bacterial communities in oral cancer lesions and those in matched controls. Thus, oral bacteria dysbiosis appears to be present during OSCC development.

Overall, five of the most abundant phyla detected in our study were consistent with those found in previous studies, specifically, *Bacteroidetes*, *Proteobacteria*, *Firmicutes*, *Fusobacteria*, and *Actinobacteria*, while the less abundant phyla detected in different surveys varied. Although *Firmicutes* was the most abundant phylum in previous studies^20–22^, *Bacteroidetes* was detected as the most abundant phylum in the current study. Of the highly abundant genera in this report, *Fusobacterium, Neisseria, Prevotella, Streptococcus, Leptotrichia, Veillonella* and *Capnocytophaga* were previously shown to be the most abundant genera in samples from periodontitis patients^31^. As opposed to *Streptococcus*, which was predominant in Pushalkar’s study^17^, *Prevotella* was the most abundant genus across all samples in our analysis, accounting for 22.5% of abundance, which is comparable to previous findings^21^. The use of HOMD enabled the assignment of many more OTUs at the species level. *Neisseria flavescens* and *Fusobacterium periodonticum* are consistently predominant in cancer lesions^23^.

Compared with other studies, our study identified larger numbers of distinguishing taxa at each level using the LEfSe method. At the phylum level, *Firmicutes* and *Actinobacteria* presented with the same patterns reported by Schmidt^21^ and were remarkably decreased in cancer lesions, while significant increases in *Fusobacteria* was also observed, consistently^24^. In line with the previous studies, genera *Streptococcus* and *Rothia* were significantly decreased in cancer lesions^21,24^. Intriguingly, the majority of these significantly enriched genera in lesions are involved in periodontal disease, including *Fusobacterium, Dialister, Peptostreptococcus, Filifactor, Peptococcus, Catonella*, and *Parvimonas* ^31^. Consistent with previous findings, remarkable enrichment of *Peptostreptococcus* and *Parvimonas* was observed in cancer samples^17,22^. Additionally, *Veillonella* was significantly decreased in cancer lesions, a finding that was previously reported in 73% of oral cancer patients after treatment^17^, indicating *Veillonella* correlates with a healthy status. Of the distinguishing species identified across the groups, forty species were highly abundant in cancer lesions, including *Porphyromonas endodontalis, Filifactor alocis* and *Dialister pneumosintes*, which are newly recognized periodontal pathogens^32^. Of all oral bacteria, *Porphyromonas gingivalis* and *Fusobacterium nucleatum* might possess the greatest potential to be correlated with oral cancer, as both have been implicated in pancreatic and colorectal cancers. Recently, a report by Gallimidi indicated *P. gingivalis* and *F. nucleatum* promote oral cancer progression via direct interactions with oral epithelial cells through Toll-like receptors^33^. However, *P. gingivalis* did not differ in abundance between groups. *Fusobacterium*, comprising the species *periodonticum, naviforme*, and *nucleatum_subsp*, was significantly enriched in lesions, accounting for 8.33%, 0.103%, and 0.297% of sequences in the cancer group, respectively. In another study, *F. periodonticum*, *F. naviforme*, and *F. nucleatum_subsp* were reported to account for 4.08%, 0.01% and 11.67% of sequences in cancer samples, respectively^24^. Thus, the different prevalences of *Fusobacterium* species detected in OSCC samples between studies may largely be due to differences in sample types, races and geographic regions of the subjects recruited. Further evidence is needed to verify these findings. A higher abundance of several *Treponema* species was observed in cancer lesions. *T. denticola*, a member of the periodontal “red complex” involved in pancreatic cancer^34^, was not included. In the literature, *Bacteroides fragilis* has been linked to colon cancer^35^, but it was not observed in our study, although it was detected in OSCC tissues in another report^23^. *Capnocytophaga* levels were significantly higher in the saliva of lung cancer patients^36^ than in healthy controls, and *Capnocytophaga gingivalis* was previously suggested to be a potential salivary biomarker of oral cancer^37^. In this study, *C. gingivalis* was detected at higher levels in control samples without any significance, while *C. leadbetteri* and *C. sp_oral_taxon_902* were remarkably overabundant in lesions. Members of the genus *Selenomonas* have been repeatedly associated with periodontal disease, although the *Selenomonas* species detected in this study did not correlate with known diseases^38^. Several species of *Peptostreptococcus* and *Parvimonas* were extensively enriched in cancer samples, including *Peptostreptococcus stomatis* and *Parvimonas micra*, both of which are reportedly related to colorectal cancer^39^. *Eikenella corrodens*, a fastidious gram-negative facultative anaerobic bacillus, was also detected in another study^24^. The genus *Eikenella* is significantly overrepresented in colorectal cancer^5^ and is associated with HPV-negative head and neck squamous cell carcinoma samples^22^. Given its documented history of pathogenicity, further investigation of the potential role of *E. corrodens* in the etiology of OSCC is warranted. In our design, paired lesion and control samples were procured from one individual, eliminating inter-individual variation. Therefore, even slight differences in the bacterial profiles between groups may be closely correlated with OSCC. Although several of the distinguishing taxa were present in relatively tiny proportions, their role in the development of OSCC should not be ignored.

Bacteria coexist in complex interaction webs, and interactions within these webs affect the species involved, while perturbations may contribute to disease^40^. As revealed in our network analysis, bacterial communities in OSCC samples presented with more complex webs depicting ecological relationships, consistent with the extensive bacterial diversity detected in the samples. The genera *Prevotella* and Neisseria clustered, forming two of the densest interaction webs in both groups. *Prevotella* and *Neisseria* may play key roles in maintaining the stability of the oral bacterial community across samples. Conversely, an association network centered around *Fusobacterium* arose in the cancer group, indicating that the genus *Fusobacterium* was implicated in the development of OSCC in accordance with its significant increase in the cancer group. *Fusobacterium* tends to co-adhere with other species in oral biofilms by forming bridges between early and late colonizers^41,42^. Thus, it was reasonable to infer a critical role for *Fusobacterium* in increasing OSCC bacterial diversity. Further evaluation of the role of *Fusobacterium* in OSCC may require more study. It was observed that the same paired taxa showed absolute opponent relationships within the groups, implicating that some drastic changes in the bacterial symbiotic relationships occurred during the oral carcinogenesis.

Functions of bacterial communities were inferred using PICRUSt based on 16 S rRNA sequence information, and differences between controls and diseased samples were further analyzed using the LEfSe method. Overall, the most abundant gene categories were replication and repair, membrane transport, amino acid metabolism, carbohydrate metabolism, and translation, likely reflecting the fundamental requirements for bacterial life in the oral mucosal habitat. As illustrated above, several gene functions exhibited remarkably different distributions within the groups. Notably, among the functions that were significantly decreased in cancer lesions, those related to membrane transport, amino acid metabolism, signal transduction, and lipid metabolism were also under-represented in periodontitis samples^43^. Periodontitis is suggested to be an independent risk factor for OSCC^44^, and bacterially induced chronic inflammation has been anticipated as being involved in oral carcinogenesis^45^. The findings observed in our study, such as several predicted functions in OSCC presenting same patterns as periodontitis and periodontitis-related taxa being significantly enriched in OSCC, indicate the pro-inflammatory potential of the bacterial communities of OSCC samples, which is consistent with a recent study^24^. Nonetheless, to obtain further details regarding the changes in gene functions of bacterial communities presenting in lesions, whole-metagenome sequencing is warranted in future studies.

In conclusion, bacterial dysbiosis was observed within OSCC surface lesion samples in our study, with drastic changes in bacterial composition and bacterial gene functions compared to controls. In particular, a group of periodontitis-correlated taxa, including *Fusobacterium, Dialister, Peptostreptococcus, Filifactor, Peptococcus, Catonella and Parvimonas*, was found to be significantly enriched in OSCC samples. In addition, several OTUs belonging to *Fusobacterium* were inferred to be heavily involved in OSCC and demonstrated good diagnostic power. The oral microbiota towards OSCC actually is considered like comorbidity factor. According to the current design and observation, it is difficult to determine whether bacterial dysbiosis changed the local microenvironment and then drove carcinogenesis or cancerization in bacterial habitats allowed bacteria suitable for a tumor microenvironment to thrive, resulting in shifts in bacterial communities. More investigation is needed.

## Methods

### Subject recruitment and sample collection

Subjects with OSCC with a median age of 62 (60% male and 40% female) were recruited from the Department of Oral and Maxillofacial-Head and Neck Oncology of the Ninth People’s Hospital (Shanghai, China). All subjects consented to clinical examination and sampling. Subjects did not have detectable periodontal inflammation, visible carious lesions, oral mucosal diseases, or any severe systemic disorders (such as diabetes, immune compromise, or genetic diseases). Moreover, they had not received treatment for OSCC or taken antibiotics at least two weeks prior to sampling. This study was approved by the Ethics Committee of Shanghai Ninth People’s Hospital affiliated with the Shanghai Jiao Tong University School of Medicine. Written informed consent was obtained from all subjects. All experiments were performed in accordance with the approved guidelines.

According to a well-defined clinical protocol, swabs of oral lesions and anatomically matched normal sites were collected. Subjects were prevented from drinking and eating for at least 2 h before sampling. All samples were transported to the laboratory on ice within 2 h of collection and were stored at −80 °C before subsequent processing.

### DNA extraction

Metagenomic DNA was individually extracted from swabs using a QIAamp DNA Mini Kit (Qiagen, Hilden, Germany) according to the manufacturer’s instructions. The quantity and quality of the isolated DNA was measured with a NanoDrop ND-1000 spectrophotometer (Thermo Fisher Scientific, Waltham, MA, USA) and by performing agarose gel electrophoresis, respectively. DNA samples were frozen at −20 °C for further analysis.

### Illumina sequencing and bioinformatics analysis of 16 S rRNA gene amplicons

Gene sequencing of 16 S rRNA was conducted on an Illumina MiSeq platform according to a previously described protocol^46^. PCR amplifications were performed with the 515 F (5′-GTGCCAGCMGCCGCGGTAA-3′) and 926 R(5′-CCGTCAATTYYTTTRAGTTT-3′) primers, which targeted the V4-V5 hypervariable region of the 16 S rRNA gene. DNA was amplified following a previously described protocol^47^. Pairs of reads from the original DNA fragments were merged using fast length adjustment of short reads (FLASH) software^48^, and sequences were analyzed using quantitative insights into microbial ecology (QIIME) software^49^. Sequences were assigned to OTUs at 97% similarity; a representative sequence was selected for each OTU, and the RDP classifier was employed to assign taxonomic data to each representative sequence.

Representative sequences were assigned at different taxonomic levels (from phylum to species) to the Human Oral Microbiome Database following the Bayesian approach with a 97% cutoff value. Bacterial diversity was determined by performing a sampling-based OTU analysis and was displayed as a rarefaction curve. Bacterial richness and diversity across samples were assessed using the following α indexes, which were estimated at a distance of 3%: Chao 1, ACE, Simpson, Shannon and Good’s coverage^50^. Student’s t-test was used to compare bacterial diversity. GraphPad Prism V.6.0 (San Diego, CA, USA) was used for graph preparation. PCA using unweighted UniFrac distance metrics was carried out, and the R package was used to visualize interactions among bacterial communities in different samples. In addition, PLS-DA, nonparametric analysis of Adonis distance matrices and ANOSIM were also performed to compare bacterial composition between samples^51^. LEfSE (http://huttenhower.sph.harvard.edu/galaxy/) was employed to identify distinguishing taxa between the two groups at multiple levels and to visualize the results using taxonomic bar charts and cladograms^52^. Network structures in the bacterial communities of the samples were defined by the Molecular Ecological Network Analysis Pipeline^53^ and visualized using Cytoscape^54^. A ROC curve was constructed to determine the diagnostic values of *Fusobacterium* OTUs for OSCC. Co-occurrence patterns for the 30 most abundant taxonomic groups across samples were explored by calculating Pearson correlation coefficients. The results were clustered and visualized using the MeV package^55^. Functional compositions of the bacterial communities were predicted using PICRUSt according to the Kyoto Encyclopedia of Genes and Genomes (KEGG) dataset^56^.

### Data Availability

The sequencing data from this study have been deposited in the GenBank Sequence Read Archive under accession number SRP097643.

## Electronic supplementary material

Supplemenary figures and legends
