# Supplementary material for: Variations in oral microbiota associated with oral cancer
Source: Sci Rep. 2017 Sep 18;7:11773. doi: 10.1038/s41598-017-11779-9 (PMC5603520; doi:10.1038/s41598-017-11779-9)

Supplementary information

Variations in oral microbiota associated with oral cancer

Hongsen Zhao[1,+], Min Chu[1,+], Zhengwei Huang[1], Xi Yang[2], Shujun Ran[1], Bin Hu[2,*] & Chenping Zhang[3,*] & Jingping Liang[1,*]

1. Ninth People's Hospital, School of Medicine, Shanghai Jiao Tong University, Department of Endodontics and Operative Dentistry, Shanghai Key Laboratory of Stomatology, Shanghai, 200011, China.

2. Ninth People's Hospital, School of Medicine, Shanghai Jiao Tong University, Department of Prosthodontics, Shanghai, 200011, China.

3. Ninth People's Hospital, School of Medicine, Shanghai Jiao Tong University, Department of Oral Maxillofacial-Head and Neck Oncology, Shanghai, 200011, China.

+ these authors contributed equally to this work

*liangjpdentist@126.com, zhang.chenping@hotmail.com, hub1093@2m9h.net

Supplementary figures

Figure S1. Box plots depict bacterial diversity differences according to the ACE (a), Chao 1 (b), and Simpson index (c) between the N and T groups. N, clinical normal samples; T, oral cancer samples.

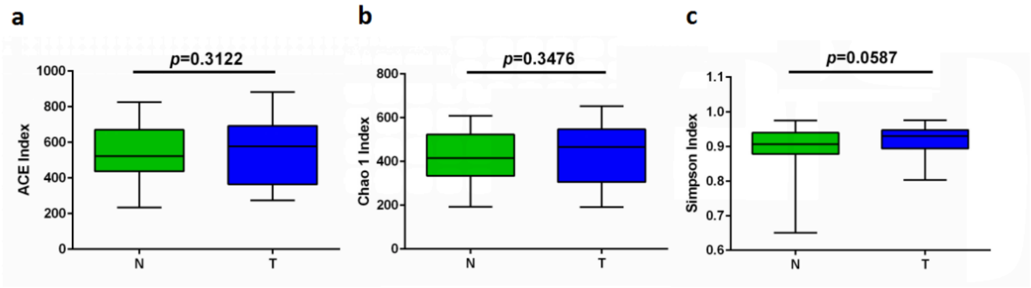

Figure S2. Classification tree of the detected species across the samples, the colored bars represent the relative abundance of the taxa. N, clinical normal samples; T, oral cancer sample.

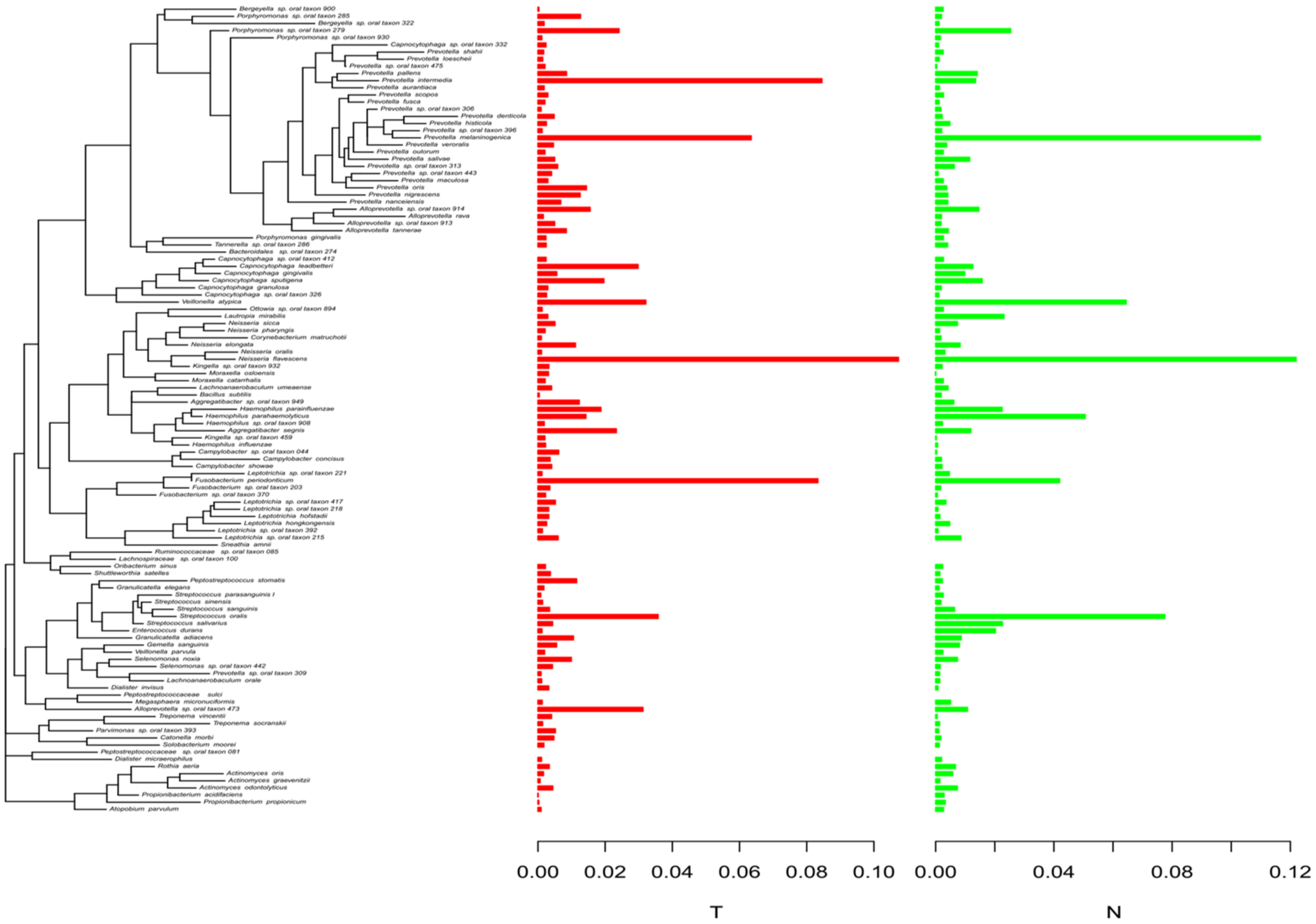

Figure S3. LDA scores showed the significant bacterial differences within the groups at species level. N, clinical normal samples; T, oral cancer samples.

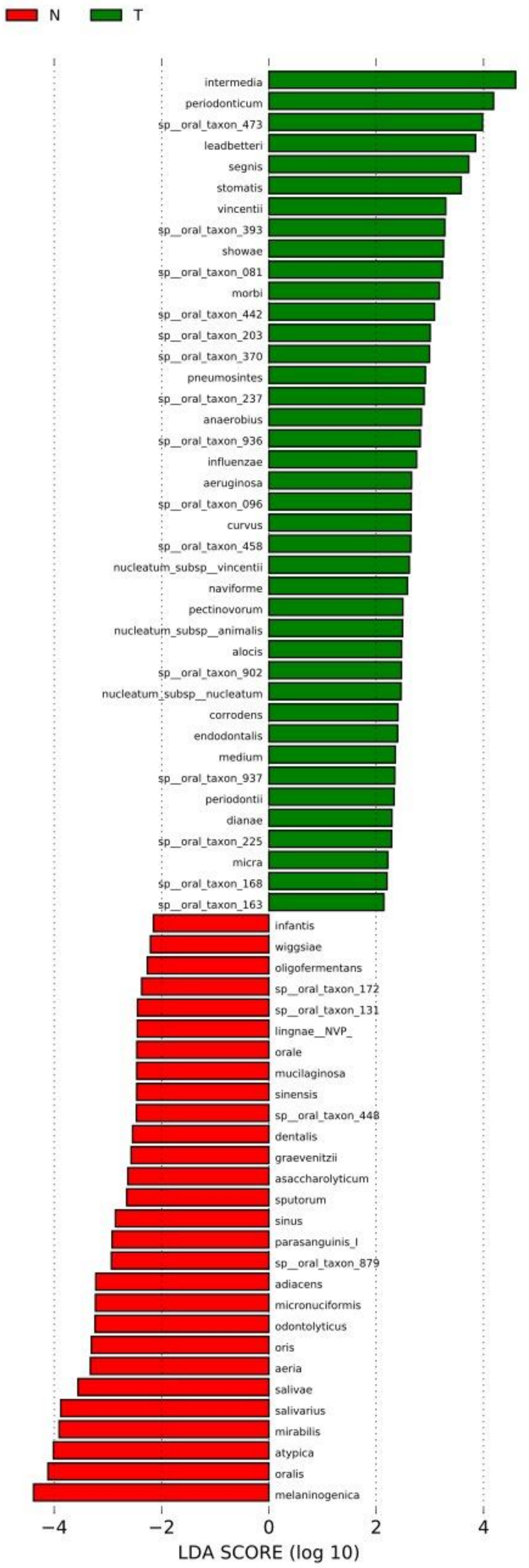

Figure S4. Network inference for the complex bacterial relationships within the N (a) and T (b) groups. Each node represented an OTU, and each edge represented a significant pairwise association (red positive, green negative) between them. The square boxes indicate the shared dense clusters in both groups, while the circle contains the highly connected bacterial cluster that formed by OTUs belonged to *Fusobacterium*, which is unique to the T group. N, clinical normal samples; T, oral cancer samples.

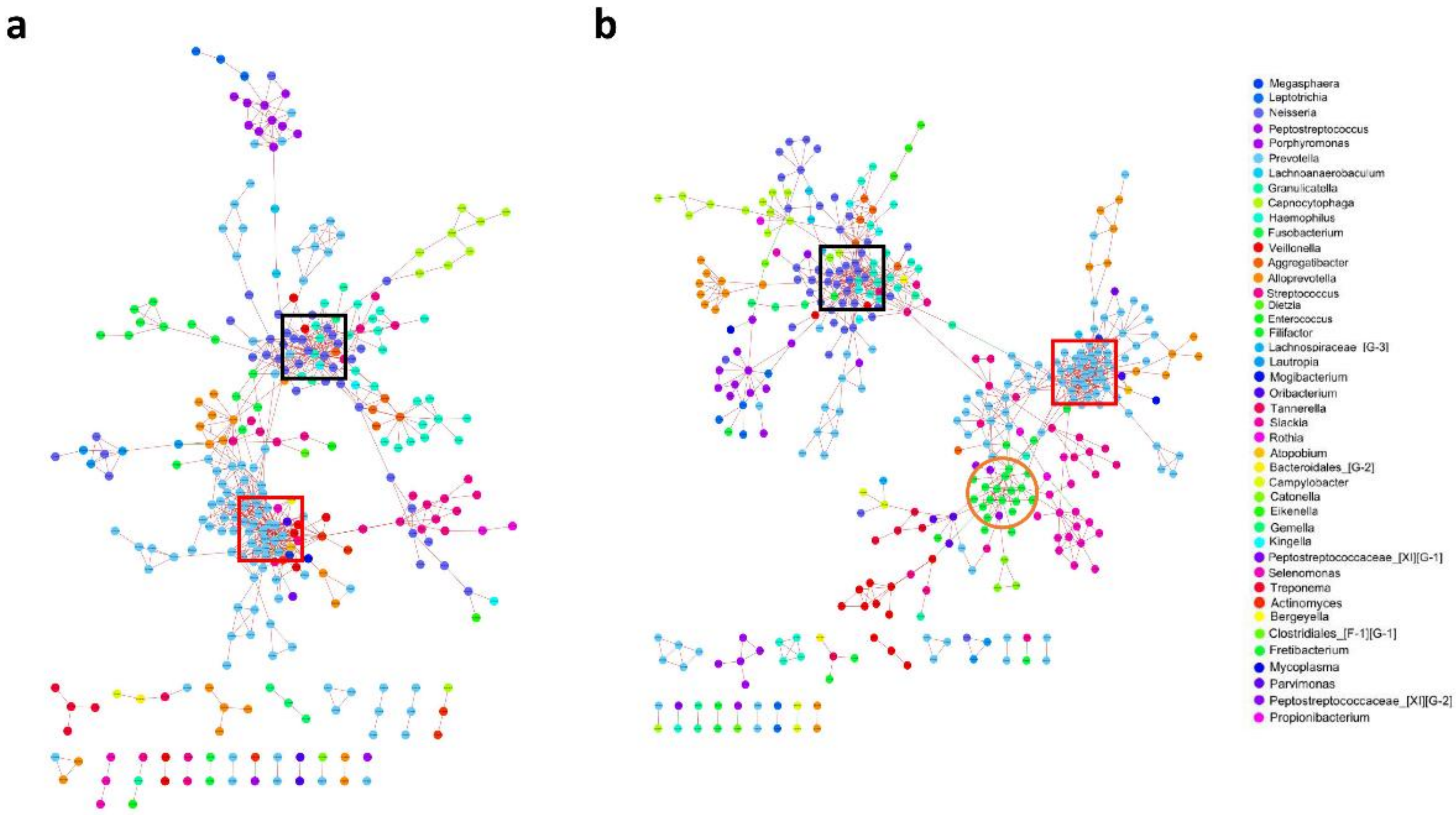

Figure S5. Co-occurrence and co-exclusion analysis of the bacterial species.

The analysis was calculated by Pearson correlations among the top 30 abundant bacterial species, and the groups were shown on the left and right, respectively. Correlation values ranged from -1.00 (green) to 1.00 (red).

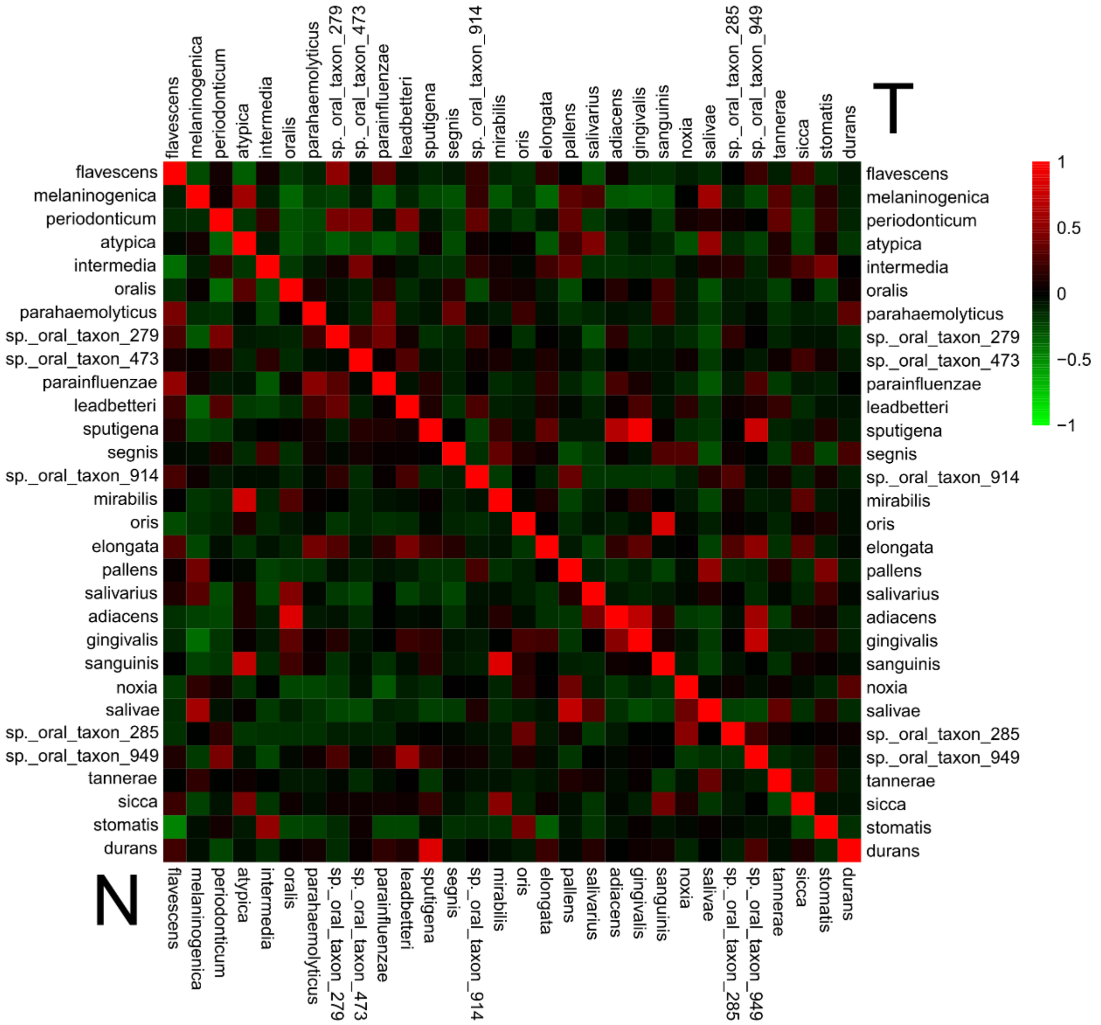

Supplement: Supplementary file 1 — Supplemenary figures and legends [file 41598_2017_11779_MOESM1_ESM.pdf]
